# A Tightly-Coupled GPS/INS/UWB Cooperative Positioning Sensors System Supported by V2I Communication

**DOI:** 10.3390/s16070944

**Published:** 2016-06-27

**Authors:** Jian Wang, Yang Gao, Zengke Li, Xiaolin Meng, Craig M. Hancock

**Affiliations:** 1 School of Environmental Science and Spatial Informatics, China University of Mining and Technology, Xuzhou 221116, China; wjianhuance@163.com (J.W.); zengkeli@cumt.edu.cn (Z.L.); 2 Nottingham Geospatial Institute, The University of Nottingham, Nottingham NG7 2TU, UK; gis_gaoyang@163.com; 3 Sino-UK Geospatial Engineering Centre, The University of Nottingham, Nottingham NG7 2TU, UK; 4 Department of Civil Engineering, University of Nottingham, Ningbo 315100, China; craig.hancock@nottingham.edu.cn

**Keywords:** cooperative positioning, GPS, INS, Robust Kalman Filter, UWB, V2I

## Abstract

This paper investigates a tightly-coupled Global Position System (GPS)/Ultra-Wideband (UWB)/Inertial Navigation System (INS) cooperative positioning scheme using a Robust Kalman Filter (RKF) supported by V2I communication. The scheme proposes a method that uses range measurements of UWB units transmitted among the terminals as augmentation inputs of the observations. The UWB range inputs are used to reform the GPS observation equations that consist of pseudo-range and Doppler measurements and the updated observation equation is processed in a tightly-coupled GPS/UWB/INS integrated positioning equation using an adaptive Robust Kalman Filter. The result of the trial conducted on the roof of the Nottingham Geospatial Institute (NGI) at the University of Nottingham shows that the integrated solution provides better accuracy and improves the availability of the system in GPS denied environments. RKF can eliminate the effects of gross errors. Additionally, the internal and external reliabilities of the system are enhanced when the UWB observables received from the moving terminals are involved in the positioning algorithm.

## 1. Introduction

Cooperative positioning is one of the key implementation techniques for the application of Intelligent Transportation Systems (ITS) and therefore this has become an area of research interest in recent years. Cooperative positioning, which is also called collaborative positioning, is a positioning approach in which a number of participants contribute their position-related information. To explain the advantage of cooperative positioning, ‘the whole is greater than the sum of its parts’ may be the best way to interpret its benefits of sharing information. In a cooperative positioning system, two or more participants are allowed to work cooperatively to improve their position performance by sharing relevant location information [1].

When a vehicle tries to obtain its own position, GPS is undoubtedly the first choice to be applied extensively due to its global coverage and ease of use. However, GPS often suffers from signal interference, multipath, and blockage, especially when a vehicle is travelling in an urban canyon. To compensate for the shortcomings of the GPS signal, extra observations transmitted from other transportation participants and sensors are desired. Aiming to improve traffic safety and enhance transportation efficiency, the U.S. Federal Communication Commission allocated 75 MHz of Dedicated Short Range Communication (DSRC) spectrum between 5.85 GHz and 5.925 GHz to be used exclusively for Vehicle to Vehicle (V2V) and Vehicle to Infrastructure (V2I) communication (referred to as V2X communication). Bridging the communication gaps among transportation participants, they are able to cooperatively position their locations by talking with surrounding participants for exchanging information via V2X communication. The authors of [2] suggest a cooperative vehicle positioning method by exchanging GPS coordinates and range measurements and the simulated results reveal that a vehicle could recognise about 70% of the surrounding vehicle with an error less than 1 m. In reference [3], researchers demonstrated a peer-to-peer cooperative localisation method by combining GNSS coordinates and terrestrial ranging measurements and the simulated 2D accuracy could achieve decimetre-level accuracy under three aiding peers cases. The authors in [4] proposed a cooperative positioning algorithm to improve localisation accuracy of vehicles by 15% using inter-vehicle distance and angle measurements in the Vehicular *Ad hoc* Network (VANET). A distributed location estimate algorithm has also been proposed to improve the positioning accuracy by extracting inter-vehicle distance from GPS pseudo-range measurements [5].

To further enhance the reliability and availability of a cooperative positioning system, many researches on multi-sensor integration and the corresponding algorithms have been performed. U.S. Federal Communications Commission established different technical standards and operating restrictions for Ultra-wideband (UWB) that UWB devices must operate in the frequency band 3.1–10.6 Hz within the measurement systems domain. Due to UWB’s large bandwidth, it has excellent multipath resolving capability, which is critical for positioning application. UWB has the capacity of penetration as well as immunity of multipath. The pseudo-range and Doppler of a Differential GPS (DGPS) and the observations of a UWB system are used as the inputs into a federated filter to perform decentralized cooperative computations for vehicles’ positions. A VANET is designed and the UWB inter-vehicle ranges were used to compensate the systematic errors [6]. In reference [7], UWB ranging was applied to augment the DGPS to improve relative positioning precision. However, all the measurements gathered from all the vehicles were used in the V2V positioning architecture. Thus its robustness and flexibility are limited due to the limitation in the communication connectivity. In reference [8], the author fused neighbours GPS position and odometer-based speed via DSRC and own GPS observation into an Extended Kalman Filter (EKF) to enhance position accuracy. It shows this method can improve the accuracy up to 48% compared with GPS positioning alone depending on the speed of the participating vehicles. Researchers in [9] invented a Pseudo-VRS solution to share RTK correction via inter-vehicle communication and the result shows that the proposed solution can help vehicle received RTK correction maintaining the availability of ambiguity fixed solutions is at least 97% as long as transmission latency is lower than 20 s. The UWB range observations are also used to augment GPS to resolve relative position between vehicles and the accuracy of baseline between vehicles could achieve better than 0.3 m in partially obstructed urban canyon [6,7]. A tightly-coupled GPS and UWB system is comprehensively investigated in [10,11,12]. The results show that the proposed system improves positioning accuracy and availability of fixed ambiguity solution especially in a GPS hostile environment.

Moreover, GPS and UWB cannot be expected to achieve 100% coverage sometimes due to their natures of line-of-sight technology, especially in urban canyon and tunnels. Inertial Navigation System (INS) has the advantage of self-contained navigation capacity that can be integrated with GPS and UWB to improve the availability and reliability of a vehicle-positioning unit. In the case that the feed of GPS and UWB observations is less than four, INS could still help to estimate the vehicle’s position. In reference [13], a loosely-coupled integration of low-cost GPS/INS and UWB solution is invented to provide about 0.2 m accuracy when both GPS and UWB observations are obtainable.

The purpose of this paper is to investigate the performance of a tightly-coupled GPS/UWB/INS cooperative positioning scheme supported by V2X communication. This paper is mainly focused on the V2V application; however, the approaches described within this paper are also applicable to V2I scenarios. The remainder of this paper is organized as follows. First of all, the application scenario of proposed scheme where a V2I communication network has been distributed is depicted in Section 2. Section 3 demonstrates the technical structure of proposed cooperative positioning scheme. Then, the Robust Kalman Filter (RKF) is presented through enumerating the observation equation involving GPS, INS and UWB measurements at the Section 4. Finally, experimental tests and discussions are described in Section 5 followed by some conclusions in Section 6.

## 2. Cooperative Positioning for V2X Application

**Figure 1:**
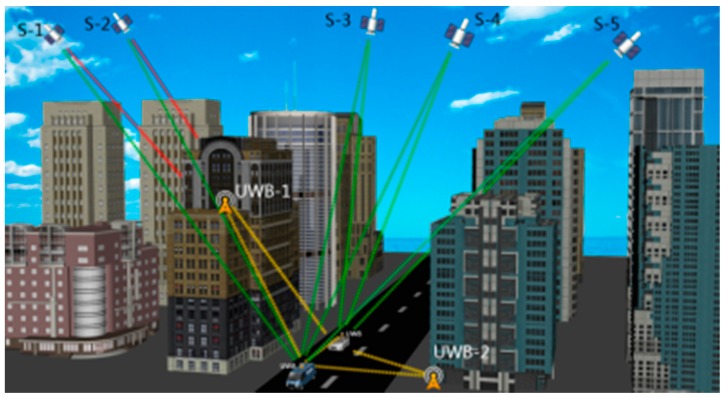
The V2X application in an urban canyon.

The V2X communication architecture as a realisation of the cooperative positioning has been developed for several years. It is assumed that a Vehicular *Ad hoc* Network (VANET) is established based on V2X communication, as shown in Figure 1. The protocol that demonstrates how a vehicle resolves its position through the proposed scheme are presented as follows: Firstly, each vehicle positioning unit calculates its own position using available data collected from both equipped sensors and surrounding vehicles via VANET, with respect to the current time. Simultaneously, the unit transmits its own measurements and the calculated position to the neighbouring vehicles. Consequentially, each vehicle positions and sends data recurrently when they are running on the road.

The V2X concept is illustrated that vehicles driven in an urban canyon are assumed to receive measurements from not only neighbouring vehicles but also infrastructure points. These data are then used to calculate the vehicles’ positions. By taking advantages of V2X communication, vehicles are able to share and exchange information with surrounding transportation participants for important safety applications, such as lane change assistance, intersection collision warning, overtaking vehicle warning, rear end collision warning, etc. [14].

However, a vehicle cannot always resolve its position when only GPS and UWB measurements are obtained from above V2X communication architecture. The reasons could be the poor performance of the GPS/UWB geometry, the limited operational range of the UWB radios and a complete blockage of GPS signals, etc. To further enhance the availability and reliability of the cooperative positioning system, an Inertial Navigation System (INS) needs to be embedded into the GPS and UWB integration positioning system and implementing algorithms need to be developed.

## 3. Tightly-Coupled GPS/UWB/INS Cooperative Positioning

**Figure 2:**
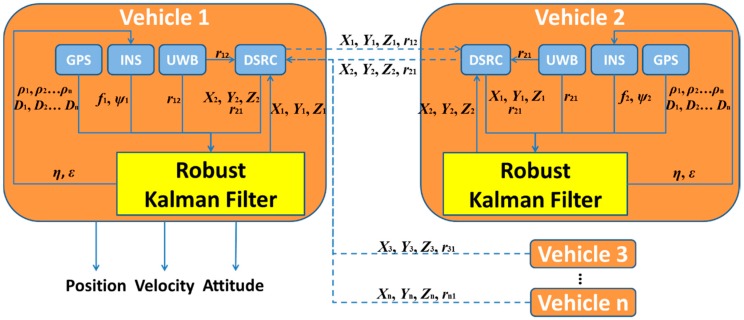
The schematic of proposed cooperative positioning method.

The schematic of the cooperative positioning scheme supported by V2I communication using GPS, UWB and INS measurements is given in Figure 2. For each vehicle positioning unit, the raw INS measurements of accelerometer and gyroscope are fed into and Robust Kalman Filter (RKF) together with UWB range measurements and the GPS coordinates of the infrastructure points and the nearby vehicles to solve the coordinate. A DSRC module is used to trade its estimated coordinate for neighbours’ coordinates and ranges with surrounding transportation participants at the beginning and end of a positioning cycle.

The RKF is applied to fuse all measurements of the multi-sensors, including pseudo-range, Doppler measurement, UWB range, acceleration and angular rate, in order to enhance the reliability of positioning capability. INS feeds its calculated pseudo-range and Doppler measurement into RFK whilst GPS and UWB also contribute their pseudo-range, Doppler measurement and range observations. Then, the accelerometer bias and gyro drift generated from RKF are fed back to the INS for next epoch calculation when position, velocity and attitude are produced. It should be mentioned that only position output is of interest here and velocity and attitude are not discussed in this paper.

## 4. Robust Kalman Filter for Cooperative Positioning

### 4.1. Dynamic Equation

The system error of the dynamics model of integrated navigation used in the Kalman Filter is designed based on the INS error equations. The insignificant terms are neglected in the process of linearization [15]. The psi-angle error equations of INS are as follows [16]:

(1) $$\delta \dot{r}=-\omega_{en}\times \delta r+\delta v$$

(2) $$\delta \dot{v}=-(2\omega_{ie}+\omega_{en})\times \delta v-\delta \psi \times f+\eta$$

(3) $$\delta \dot{\psi }=-(\omega_{ie}+\omega_{en})\times \delta \psi +\varepsilon$$

where δr, δv and δψ are the position, velocity and orientation error vectors, respectively. $\omega_{en}$ is the rate of navigation frame with respect to earth, and $\omega_{ie}$ is the rate of earth with respect to inertial frame. Besides, ***f*** is the force observation. Then, the system error dynamics of GPS/INS integration is obtained by expanding the accelerometer bias error vector η and the gyro drift error vector ε.

The accelerometer bias error vector η and the gyro drift error vector ε are regarded as the random walk process vectors, which are modelled as follows [17]:

(4) $$\dot{\eta }=u_{\eta }$$

(5) $$\dot{\varepsilon }=u_{\varepsilon }$$

where $u_{\eta }$ and $u_{\varepsilon }$ are white Gaussian noise vectors.

The receiver clock state dynamic equations can be written as follows:

(6) $$\dot{d}t=\delta dt+u_{dt}$$

(7) $$\delta \dot{d}t=u_{\delta dt}$$

where $u_{dt}$ and $u_{\delta dt}$ are white Gaussian noise vectors of receiver clock error and receiver clock error drift.

By combining Equations (1) and (7), the system dynamics model can be generalized in matrix and vector form [18]:

(8) $$\dot{X}=FX+u$$

where ***X*** is the error state vector which contains position error, velocity error, attitude error, accelerometer bias, gyro drift, clock error and clock drift. ***F*** is the system transition matrix, and ***u*** is the process noise vector.

### 4.2. Observation Equation

The observation model in GPS/INS/UWB tightly-coupled positioning scheme is composed of the pseudo-range and Doppler difference vector among the UWB, GPS observation and the INS computation value [19]:

(9) $$Z=\left[\begin{array}{c} P_{j}^{\text{GPS}}-P_{j}^{\text{INS}}\\ D_{j}^{\text{GPS}}-D_{j}^{\text{INS}}\\ r_{i}^{\text{UWB}}-r_{i}^{\text{INS}}\\ \vdots \end{array}\right]$$

where $P_{j}^{\text{GPS}}$ and $D_{j}^{\text{GPS}}$ are the pseudo-range and Doppler value observed by the *j*th GPS satellite, respectively; $P_{j}^{\text{INS}}$ and $D_{j}^{\text{INS}}$ are the pseudo-range and Doppler measurement of the *j*th satellite predicted by INS, respectively; $r_{i}^{\text{INS}}$ is the space distance calculated via the three-dimensional coordinates of a moving vehicle estimated using a strap down inertial system algorithm and the three-dimensional coordinates of the UWB reference stations and $r_{i}^{\text{UWB}}$ is the UWB range measurements of the *i*th UWB unit [20]:

(10) $$r_{i}^{\text{UWB}}=\sqrt{{\left(x^{\text{UWB}}-x\right)}^{2}+{\left(y^{\text{UWB}}-y\right)}^{2}+{\left(z^{\text{UWB}}-z\right)}^{2}}$$

where $x^{\text{UWB}}$, $y^{\text{UWB}}$ and $z^{\text{UWB}}$ are the three-dimensional coordinates of the UWB reference stations and *x*, *y*, *z* are the three-dimensional coordinates of a moving vehicle to be solved.

The generic measurement equation system of the Kalman Filter can be written as:

(11) $$Z_{k}=H_{k}X_{k}+\tau$$

where $H_{k}$ is the observation matrix [20], and τ is the measurement noise vector with covariance matrix $R_{k}$, assumed to be white Gaussian noise.

### 4.3. Robust Kalman Filter

The optimal estimates of the state vector from the Kalman Filter can be reached through a time update and an observation update. The time update process of Kalman Filter is independent, which is written as:

(12) $${\bar{X}}_{k}=F_{k,k-1}{\hat{X}}_{k-1}$$

(13) $${\bar{C}}_{k}=F_{k,k-1}C_{k-1}F_{k,k-1}^{T}+Q_{k-1}$$

The observation update equation of Kalman Filter is expressed as:

(14) $$V_{k}=Z_{k}-H_{k}{\bar{X}}_{k}$$

(15) $${\hat{X}}_{k}={\bar{X}}_{k}+G_{k}V_{k}$$

(16) $$G_{k}={\bar{C}}_{k}H_{k}^{T}{\left(H_{k}{\bar{C}}_{k}H_{k}^{T}+\frac{1}{\gamma_{k}}R_{k}\right)}^{-1}$$

(17) $$C_{k}=\left(I-G_{k}H_{k}\right){\bar{C}}_{k}$$

where ${\bar{X}}_{k}$ is a priori state estimation, ${\hat{X}}_{k}$ is a *posteriori* state estimation, $G_{k}$ is the gain matrix of Kalman Filter, ${\bar{C}}_{k}$ is a priori covariance matrix of state vector, $C_{k-1}$ is a *posteriori* covariance matrix of state vector, $R_{k}$ is the covariance matrix of measurement noise vector, $Q_{k-1}$ is the covariance matrix of process noise, and $F_{k,k-1}$ is the system transition matrix from epoch k − 1 to epoch k, $\gamma_{k}$ is the robust factor. The robust factor $\gamma_{k}$ is calculated by the IGGIII scheme to improve calculation efficiency through a piecewise function. A different robust factor is set according to the value of the gross error [21,22]:

(18) $$\gamma_{k}=\left\{ \begin{array}{l} 1,s_{k}\leq k_{0}\\ \frac{k_{0}}{s_{k}}\times {\left[\frac{k_{1}-s_{k}}{k_{1}-k_{0}}\right]}^{2},k_{0}<s_{k}\leq k_{1}\\ 10^{-30},s_{k}>k_{1} \end{array}\right.$$

where *k*_0_ and *k*_1_ are constants which have the values *k*_0_ = 2.5~3.5 and *k*_1_ = 3.5~4.5, respectively.

(19) $$s_{k}=V_{k}/\left(\sigma_{k}\sqrt{d_{k}}\right)$$

where *V_k_* and *d_k_* are the predicted residual and their reciprocal of the weight of the observation vector, $\sigma_{k}$ the variance of the unit weight can be written as:

(20) $$\sigma_{k}=1.4826\times MEDIAN\left(\left|V_{k}\right|/\sqrt{d_{k}}\right)$$

This section may be divided by subheadings. It should provide a concise and precise description of the experimental results, their interpretation, as well as the experimental conclusions that can be drawn.

## 5. Experimental Tests

Field tests were conducted to evaluate the performance of the proposed scheme on the roof of the Nottingham Geospatial Institute (NGI) [23]. The test consists of one tactical grade Inertial Measurement Unit (IMU), 3 UWB units, and 4 GPS receivers. The sampling rate of the UWB unit was 1 Hz and its ranging error will be evaluated in following section using a stochastic analysis model. The specification of the IMU is given in Table 1.

**Figure 3:**
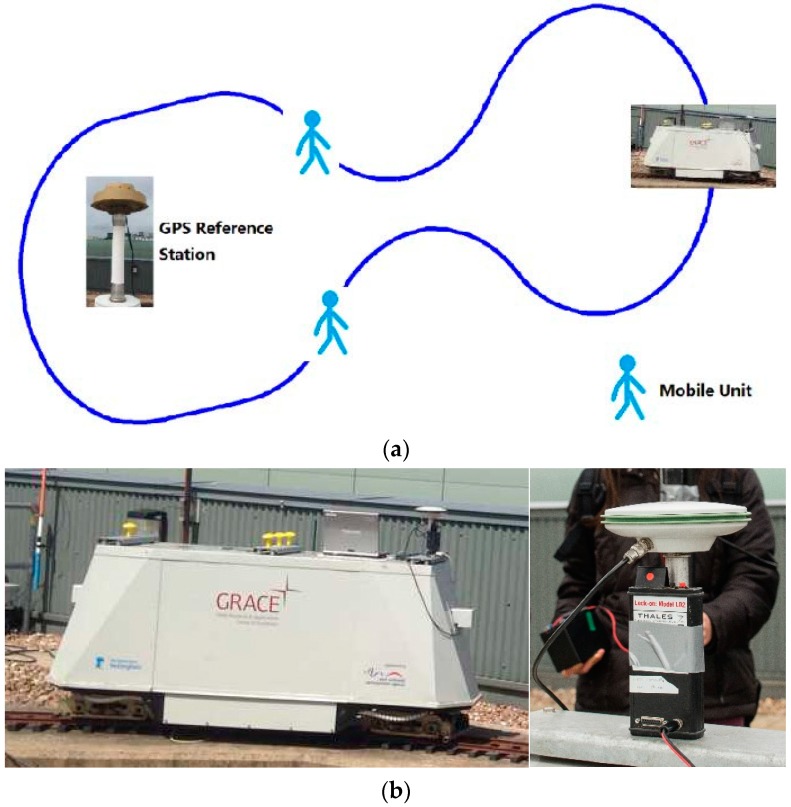
The test scenario for verifying the proposed architecture. (**a**) Test deployment diagram; (**b**) the locomotive and GPS and the UWB unit.

In the beginning, a Leica AS10 GNSS dual-frequency antenna was installed on the top of a pillar above the NGI locomotive and a UWB unit was fastened under the antenna with a known lever-arm. This UWB unit was also connected to a laptop to store the range observations between it and other two moving UWB units. The SPAN IMU inside the locomotive was connected to the Leica antenna and recorded raw observations into a SD card for post-processing. Then two people walked along the track and each of them was equipped with a set of equipment that included a GPS receiver, a UWB unit and a battery. The product type of the UWB unit is Lock-on Model LD2 provided by Thales Research & Technology Ltd., West Sussex, UK. The unit complies with the standard UWB power density limit of −41.3 dBm/MHz, which leads to a nominal output power of 100 μW. The transmission is Frequency Hopped Direct Sequence Spread Spectrum signal covering: 4760 MHz to 6200 MHz. The range accuracy of decimetre level can be achieved by UWB. To calibrate the GPS and the UWB antenna precisely, the GPS pillar fastened with UWB was held vertically as stable as possible during the tests. Another GPS receiver was set on one of the pillars on the NGI roof to act as the reference station. As the UWB ranges were time tagged by the laptop system time, the laptop was synchronized to GPS time to collect 1 Hz UWB ranges prior to the test. Before starting the tests, we assume that the locomotive and two people were carrying a DSRC device within the reachable communication range. The sampling rate of GPS receivers and IMU were respectively configured as 10 Hz and 200 Hz to simulate a running vehicle under V2I communication environment. The locomotive ran along the track to act as a vehicle that is able to exchange raw observations and distributed positioning coordinates with surrounding traffic participants (thee two people). Therefore, a V2I network consists of a running vehicle, two participants and a reference station is plotted in Figure 3a and the devices used during the tests are presented in Figure 3b.

**Figure 4:**
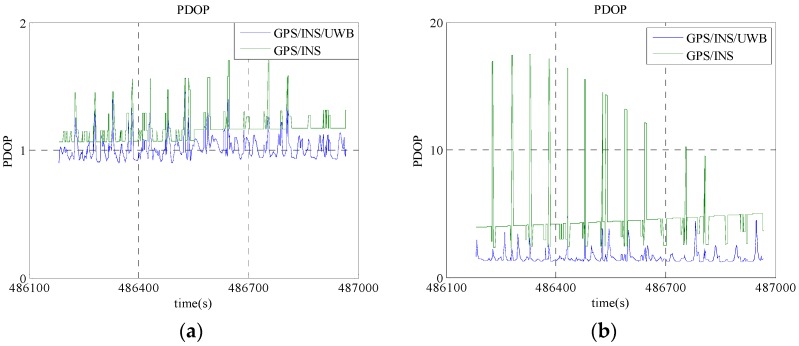
The DOP value variations during the experimental period. (**a**) In the case of more than four GPS satellite; (**b**) in the case of four GPS satellite.

A GPS software tool called GrafNav^TM^ (Version 8.0) is used to post-process the ambiguity-fixed GPS RTK solution to obtain the trajectory of locomotive for verification purpose and the achievable accuracies with post-processing are listed in Table 2. The DOP value variation during the experimental period is illustrated in Figure 4. In the case that more than 4 visible GPS satellites shown in Figure 4a, the PDOP value of the GPS only case is greater than 1 and the maximum value approaches 2, while the PDOP value of the cooperative positioning by including UWB range is approximately 1 throughout the experiment. When the number of the GPS satellites drops to 4, the PDOP value almost approaches to 20 while the PDOP value of the cooperative positioning still remains less than 5 as shown in Figure 4b. It is illustrated that the fusion of the GPS/UWB/INS raw observations obtained via V2I communication network can greatly improve the reliability of positioning capability.

To overall evaluate the performance of the cooperative positioning scheme, six scenarios are considered. The average velocity of the moving platform from all experience is about 2 m/s:

Scenario 1: Integration of GPS/INS using pseudo-ranges, Doppler observations and raw INS observations for cooperative positioning in the case of more than four satellites.
Scenario 2: Integration of GPS/UWB/INS using pseudo-ranges, Doppler observations, UWB range and raw INS observations for cooperative positioning in the case of more than four satellites.
Scenario 3: Integration of GPS/INS using pseudo-ranges, Doppler observations and raw INS observations for cooperative positioning in the case of only four satellites.
Scenario 4: Integration of GPS/UWB/INS using pseudo-ranges, Doppler observations, UWB ranges and raw INS observations for cooperative positioning in the case of only four satellites.
Scenario 5: Integration of GPS/UWB/INS using pseudo-ranges, Doppler observations, UWB ranges and raw INS observations for cooperative positioning in the case of three, two and one visible satellites.
Scenario 6: Integration of GPS/UWB/INS using pseudo-ranges, Doppler observations, UWB ranges and raw INS observations in the case of gross errors.

### 5.1. Stochastic Model of UWB Ranges

**Figure 5:**
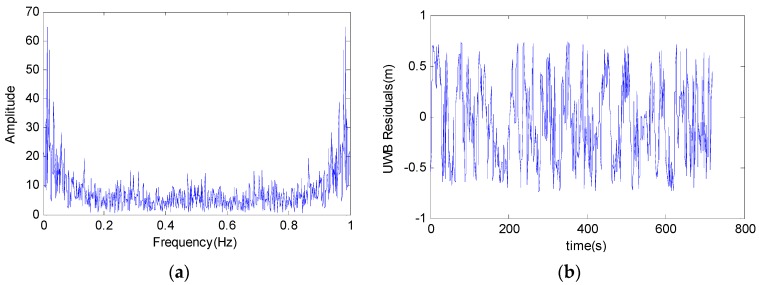
The residuals of the UWB measurement and its frequency analysis result. (**a**) Frequency characteristics of the UWB signal; (**b**) the residuals of the UWB measurement.

To obtain the stochastic parameters of the UWB measurements, autocorrelation function and probability density function are analysed in an off-line manner. The residuals of a UWB data series of 718 epochs with a frequency of 1 Hz are obtained via subtracting UWB range with the real distance that are measured with a total station. The frequency analysis, shown in Figure 5, demonstrates little information of the UWB raw measurements. The unbiased estimate of the autocorrelation coefficient is calculated using autocorrelation function method, and also the probability density function of the residuals is estimated. As shown in Figure 5a, the delay of the autocorrelation estimate of the predicted residuals is zero and the variance of the residual is 0.14 m^2^. In Figure 5b, the absolute values of the residuals of the UWB measurements are all less than 0.2 m that proves the relationship among the data is low.

**Figure 6:**
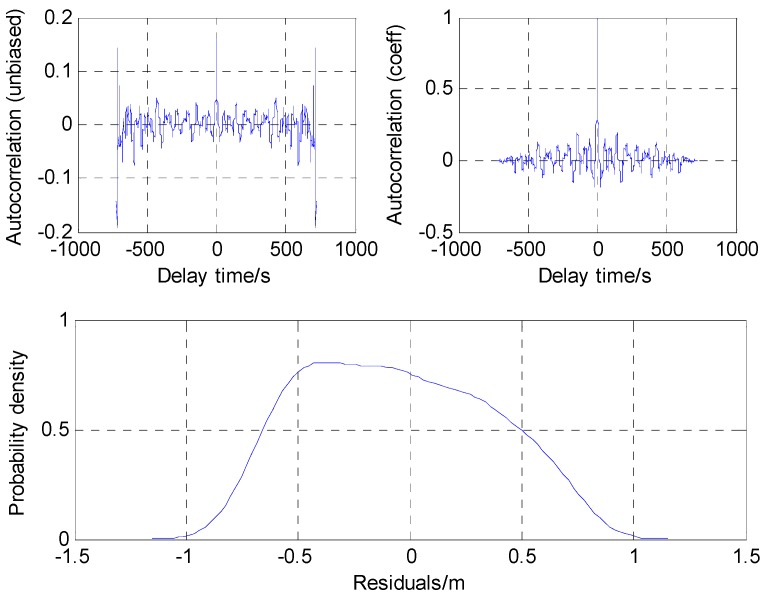
Auto-correlation function and probability density function of residuals.

As shown in Figure 6, the predicted residuals approximately coincide with the Gaussian distribution and the variance of 0.14 m^2^ is then used as the value of the stochastic parameter in the mentioned Robust Kalman Filter (RKF) model.

According to Equation (8), the stochastic models of the parameters including position, velocity and orientation, accelerometer bias and gyro drift are influenced by accelerometer and gyro observation. Thus, the IMU, which was fixed in a three-axis rotary table, was tested and the observation was analysed using the autocorrelation analysis to obtain the spectral density. There was difference of the spectral density for the accelerometer and gyro observation between in the lab environment and in the field test, so the value of stochastic model was set larger than the test value according to experience. In the data processing, the parameters of the stochastic model of the dynamic model were determined based on experience. The initial position errors were 1.5 m, 1.5 m, and 3 m and the initial velocity errors were 0.2 m/s, 0.2 m/s, and 0.8 m/s in NED directions, respectively. The initial platform alignment errors were 0.1°, 0.1°, and 1°. According to the measurement accuracy of IMU, the initial standard deviations of gyro and accelerometer biases were 15°/h and 600 mg, respectively.

### 5.2. GPS/INS/UWB Cooperative Positioning

**Figure 7:**
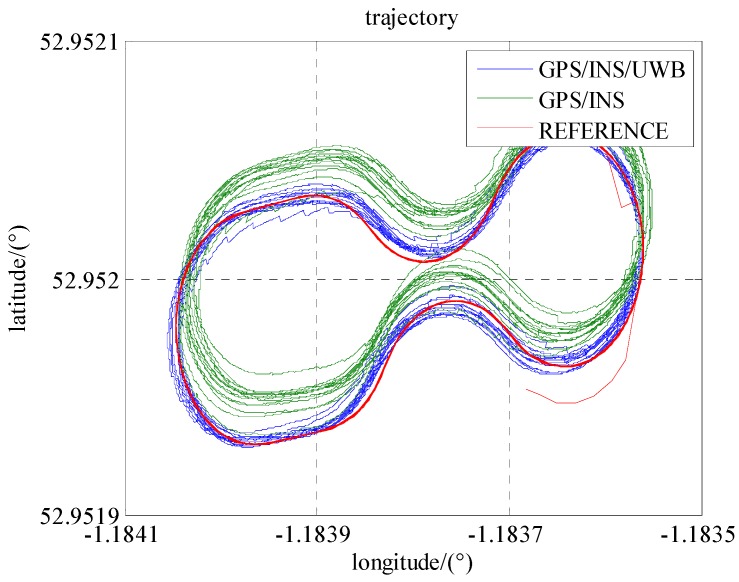
The trajectories of the locomotive in Scenario 1 and 2.

**Figure 8:**
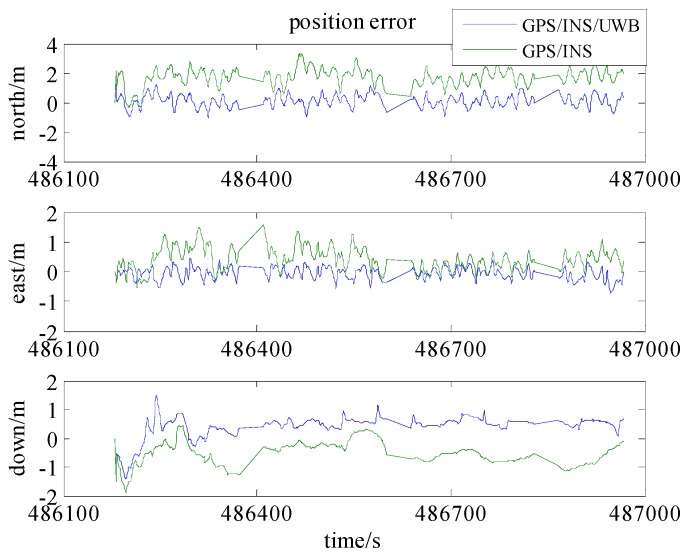
The position accuracies in Scenario 1 and 2.

As seen in Figure 7, the trajectory of GPS/INS integration has a systematic bias mainly caused by the errors in the pseudo-range and Doppler observations. The reasons for this could be the satellite obit errors, clock errors atmospheric errors, etc. Figure 8 and Table 3 show that the positioning accuracies (RMS) in the north, east and down are improved 76%, 61%, and 16%, respectively.

**Figure 9:**
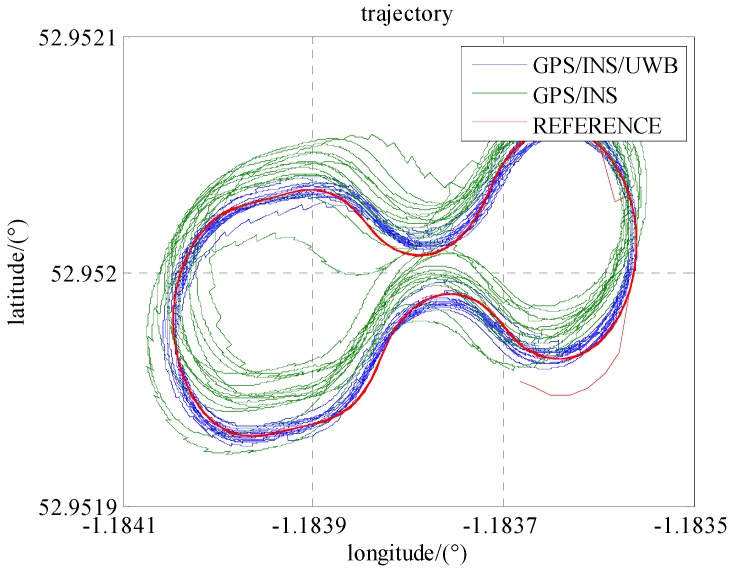
The trajectories of the locomotive in Scenario 3 and 4.

**Figure 10:**
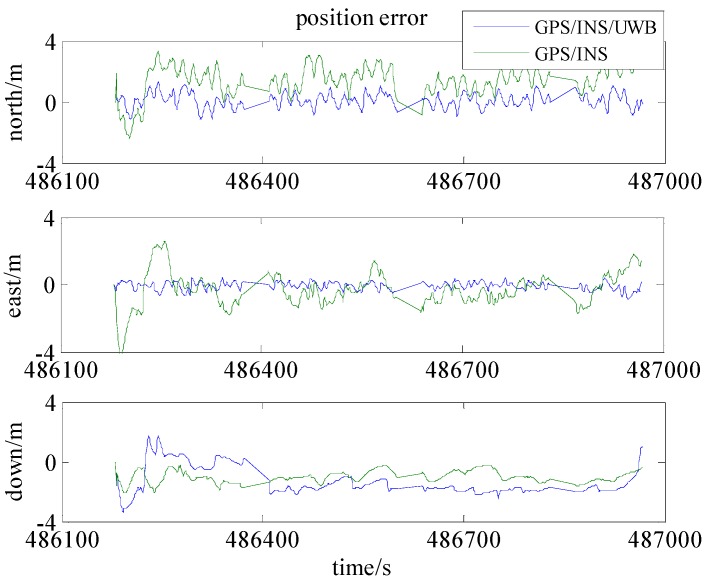
The position accuracies in Scenario 3 and 4.

In the case of Scenario 3 and 4, i.e., only four visible GPS satellites, the comparison between those two scenarios is shown in Figure 9. It is similar to Figure 7 in that the systematic error has been removed by integrating UWB observations. By integrating UWB range measurements, the cooperative positioning accuracies in north, east and down are improved by 73%, 76%, and 33%, respectively, in Figure 10 and Table 4. It is also noticed that the improvements of accuracy in the case of only 4 satellites are superior to the case of more than 4 satellites after integrating the UWB observation.

### 5.3. GPS/INS/UWB Cooperative Positioning in GPS Denied Environments

**Figure 11:**
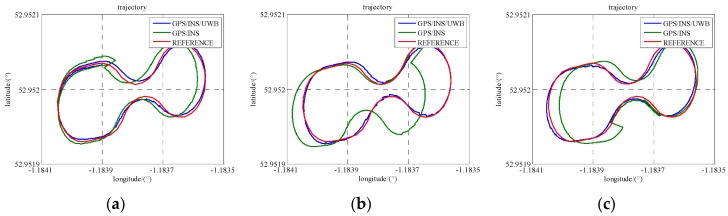
The trajectories of the locomotive in three GPS blockage periods (Scenario 5). (**a**) one gap with three satellites; (**b**) one gap with two satellites; (**c**) one gap with one satellite.

**Figure 12:**
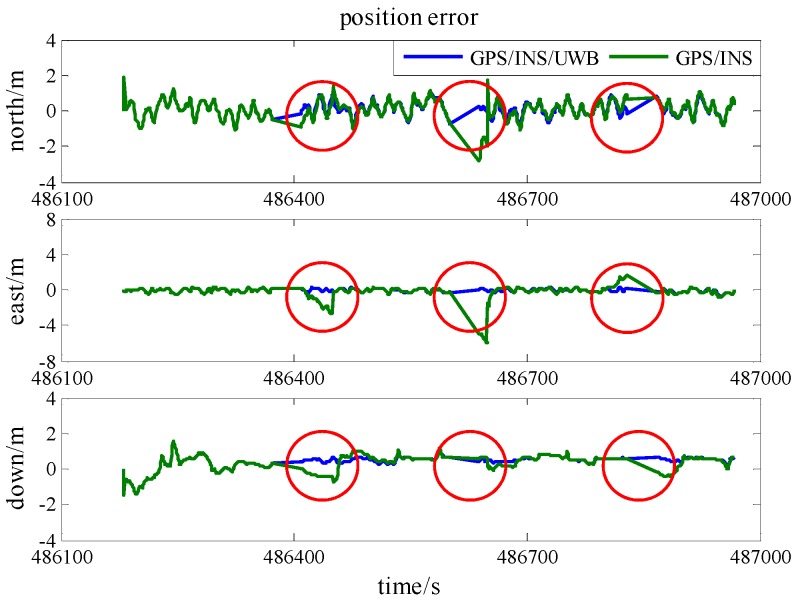
The position accuracies in three GPS blockage periods (Scenario 5).

In Scenario 5, to verify the performance of the cooperative positioning scheme, three gaps of GPS blockage are manually created. Three satellites are reserved between 486,400 s and 486,450 s, two satellites are reserved between 486,600 s and 486,650 s, and just one satellite is reserved between 486,800 s and 486,850 s. The trajectories of three gaps are illustrated in Figure 11 and the position error is demonstrated in Figure 12. In the case of less than 4 satellites, the positioning errors enlarge with time and the INS errors cannot be estimated correctly. The integration of UWB range measurement can drag the trajectory back to the right way and remain relative high accuracy. As listed in Table 5, GPS/INS/UWB solution gives half-meter accuracies in the north and east directions during three periods while the accuracies of GPS/INS solution are beyond one meter. As the locomotive was running on a flat track, the accuracy of height does not drift away a lot because of the loss of the GPS satellite. In addition, the accuracies in the north, east and height directions gradually degrade on account of decreasing visible GPS satellite.

### 5.4. Robustness Test of Robust Kalman Filter

**Figure 13:**
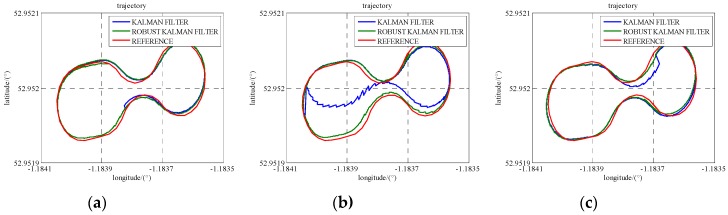
The trajectories with an added GPS gross error using standard Kalman Filter and RKF. (**a**) one gap at the times of 486,420 s; (**b**) one gap at the times of 486,640 s; (**c**) one gap at the times of 486,820 s.

**Figure 14:**
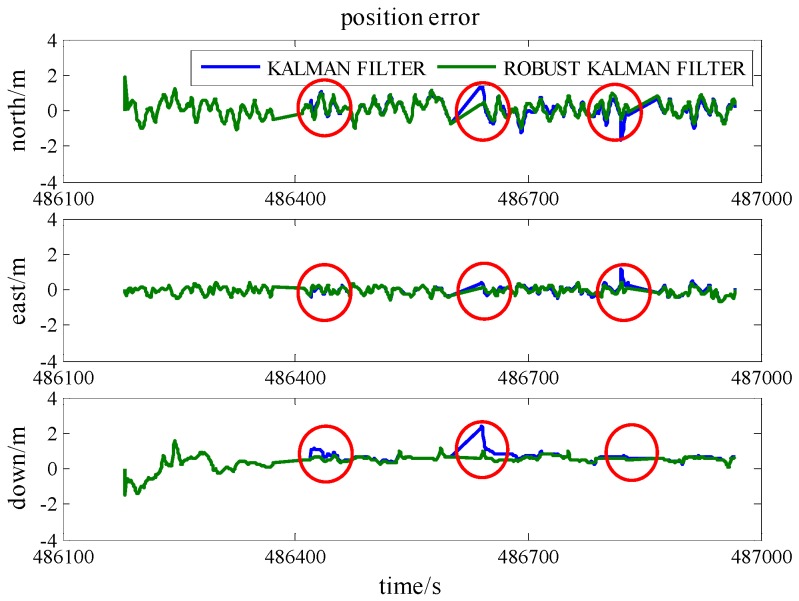
The position accuracy comparison between standard Kalman Filter and RKF with an added pseudo-range gross error.

To demonstrate the robustness of the model, simulated gross errors were added to the GPS and UWB observations at the times of 486,420 s, 486,640 s and 486,820 s under Scenario 6. The trajectories shown in Figure 13 demonstrate the effect of a gross error of 20 m added onto SV 27 pseudo-range measurement and Figure 14 reveals the corresponding positioning error. The good shapes of green trajectories prove that the effect of added gross error is eliminated by using RKF. As listed in Table 6, RKF improves accuracies of 70%, 62%, and 19%, at epoch 486,820 s in the north, east and height directions, respectively, comparing against the accuracies of standard Kalman Filter.

**Figure 15:**
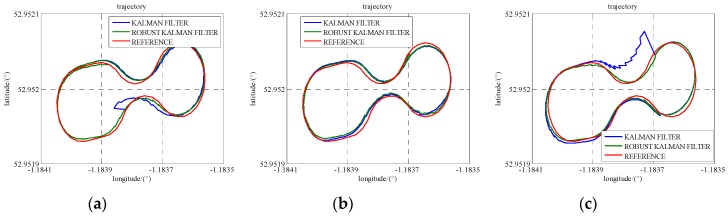
The trajectories with an added UWB gross error using standard Kalman Filter and RKF. (**a**) one gap at the times of 486,420 s; (**b**) one gap at the times of 486,640 s; (**c**) one gap at the times of 486,820 s.

**Figure 16:**
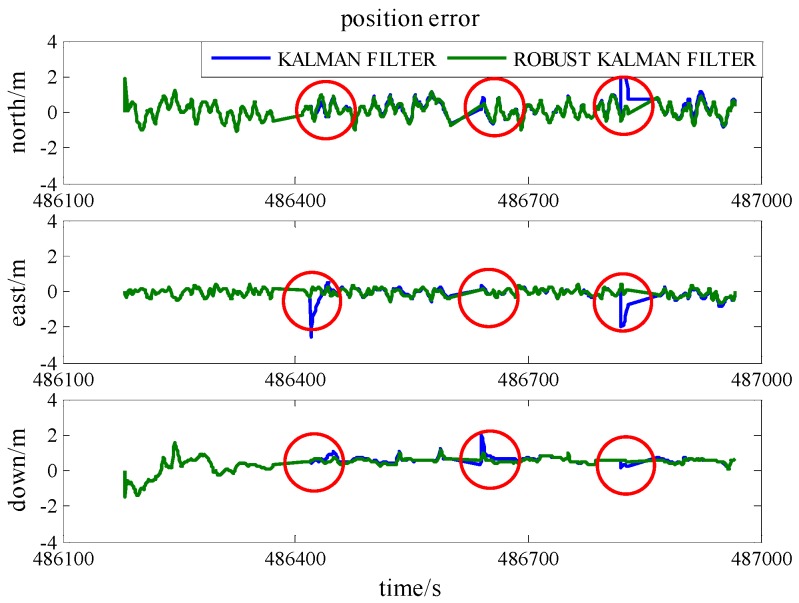
The position accuracy comparison between standard Kalman Filter and RKF with an added UWB ranging gross error.

Figure 15 reveals the trajectories calculated when a gross error of 2 m is added onto a UWB range measurement and the corresponding position error is illustrated in Figure 16. At epoch 486,640 s in Table 7, RKF gives 0.40 m, 0.16 m, and 1.00 m accuracies in the north, east and height directions, respectively, as well as they are improvements of 23%, 50%, and 49% compared with the result of standard Kalman Filter. As a result, RKF not only limits the effect of pseudo-range gross error, but also works fine on the UWB ranging gross error.

## 6. Conclusions

This paper has proposed a cooperative positioning scheme supported by V2I communication. A tightly-coupled GPS/UWB/INS algorithm based on a Robust Kalman Filter (RKF) is illustrated for the implement of proposed cooperative positioning scheme. Additionally, a gross error detection procedure is embedded at the beginning of RKF which reliefs the calculation burden of the algorithm. Six different scenarios are simulated to comprehensively evaluate the performance of the algorithm. It is shown that the compensation of UWB into GPS/INS can improve the reliability and precision of the cooperative positioning scheme and also could achieve overall sub-metre accuracy. Particularly, UWB range measurement remains the positioning availability when there are less than four visible GPS satellites. Therefore, the characteristics of kinematic communication node should be examined.

Positioning with multi-sensor integration has achieved encouraging results in recent years. However, it cannot always meet the requirements particularly in the urban canyon environment. Benefiting from the emerging of V2X communication, cooperative positioning opens another way to support numerous V2X applications. A cooperative positioning scheme supported by V2I communication (i.e., stationary node) has been proposed in the present paper and this method will be extended to V2V communication further to V2X communication. The GPS carrier-phase integration and more sensory sources are considered as the future work. At the same time, more sensors mean more redundant information. Useful information selecting and efficient filtering algorithms are very key issues for multi-sensor fusion.

## Figures and Tables

**Table 1:** Tactical grade IMU technical specifications.

Parameters	Gyroscope	Accelerometer
Bias	1°/h	1.0 mg
Scale factor	150 ppm	300 ppm
Random walk	0.125°/sqrt(h)	6 mg/sqrt(Hz)
Sampling Rate	200 Hz	

**Table 2:** GPS accuracy with post-processing.

Scenarios	Horizontal	Vertical
static	5 mm + 0.5 ppm	10 mm + 0.5 ppm
kinematic	10 mm + 1 ppm	20 mm + 1 ppm

**Table 3:** Comparison of Scenario 1 and 2 in terms of position error.

Scenario	RMS (m)			MAX (m)		
	North	East	Down	North	East	Down
1	1.82	0.57	0.68	3.30	1.60	1.89
2	0.44	0.22	0.57	1.86	0.70	1.53

**Table 4:** Comparison between Scenario 3 and 4 in terms of position error.

Scenario	RMS (m)			MAX (m)		
	North	East	Down	North	East	Down
3	1.75	1.09	1.64	3.29	4.59	3.37
4	0.48	0.26	1.10	1.86	0.87	2.15

**Table 5:** Position errors in three insufficient GPS satellites periods.

Gaps	GPS/INS/UWB (m)			GPS/INS (m)		
	North	East	Down	North	East	Down
486400s–486450s	0.42	0.17	0.39	0.56	1.34	0.39
486600s–486650s	0.41	0.18	0.53	1.03	2.65	0.54
486800s–486850s	0.46	0.17	0.58	0.54	0.92	0.58

**Table 6:** Comparison between standard Kalman Filter and RKF in term of positioning accuracy with an added pseudo-range gross error.

Epochs	Standard KF (m)			RKF (m)		
	North	East	Down	North	East	Down
486420s	0.54	−0.37	1.11	0.22	−0.10	0.57
486640s	1.26	0.44	2.38	0.30	0.11	0.78
486820s	−1.66	1.17	0.70	−0.49	0.44	0.57

**Table 7:** Comparison between standard Kalman Filter and RKF in term of positioning accuracy with an added UWB ranging gross error.

Epochs	Standard KF (m)			RKF (m)		
	North	East	Down	North	East	Down
486420s	0.34	−2.54	0.42	0.22	−0.10	0.57
486640s	0.52	0.32	1.96	0.40	0.16	1.00
486820s	3.36	−1.97	0.20	−0.49	0.44	0.57

## References

[1] Garello R., Lo Presti L., Corazza G., Samson J. Peer-to-Peer Cooperative Positioning Part I: GNSS Aided Acquisition. http://www.insidegnss.com/auto/marapr12-WP.pdf.

[2] Fujii S., Fujita A., Umedu T., Kaneda S., Yamaguchi H., Higashino T., Takai M. Cooperative vehicle positioning *via* V2V communications and onboard sensors. Proceedings of the 2011 IEEE Vehicular Technology Conference.

[3] Deambrogio L., Palestini C., Bastia F., Gabelli G., Corazza G.E., Samson J. Impact of high-end receivers in a peer-to-peer cooperative localization system. Proceedings of the International Conference on Ubiquitous Positioning, Indoor Navigation and Location-Based Service.

[4] Tsai M.-F., Wang P.-C., Shieh C.-K., Hwang W.-S., Chilamkurti N., Rho S., Lee Y.S. (2014). Improving positioning accuracy for VANET in real city environments. J. Supercomput..

[5] Liu K., Lim H.B. Positioning accuracy improvement via distributed location estimate in cooperative vehicular networks. Proceedings of the 2012 15th International IEEE Conference on Intelligent Transportation Systems.

[6] Wang D., O’Keefe K., Petovello M.G. Decentralized cooperative navigation for vehicle-to-vehicle (V2V) applications using GPS integrated with UWB range. Proceedings of the ION Pacific PNT 2013 Conference.

[7] Petovello M.G., O’Keefe K., Chan B., Spiller S., Pedrosa C., Xie P., Basnayake C. (2012). Demonstration of inter-vehicle UWB ranging to augment DGPS for improved relative positioning. J. Glob. Position. Syst..

[8] Alam N., Balaei A.T., Dempster A.G. (2011). A DSRC Doppler-based cooperative positioning enhancement for vehicular networks with GPS availability. IEEE Trans. Veh. Technol..

[9] Stephenson S., Meng X., Moore T., Baxendale A., Edwards T. A fairy tale approach to cooperative vehicle positioning. Proceedings of the 2014 International Technical Meeting of The Institute of Navigation (ION ITM 2014).

[10] Chiu D.S., MacGougan G., O’Keefe K. UWB assisted GPS RTK in hostile environments. Proceedings of the ION NTM 2008.

[11] MacGougan G., O’Keefe K., Chiu D. Multiple UWB range assisted GPS RTK in hostile environments. Proceedings of the 21st International Technical Meeting of the Satellite Division of The Institute of Navigation (ION GNSS 2008).

[12] MacGougan G., O’Keefe K., Klukas R. (2010). Tightly-coupled GPS/UWB integration. J. Navig..

[13] Tanigawa M., Hol J.D., Dijkstra F., Luinge H., Slycke P. Augmentation of low-cost GPS/MEMS INS with UWB positioning system for seamless outdoor/indoor positioning. Proceedings of the 21st International Technical Meeting of the Satellite Division of The Institute of Navigation (ION GNSS 2008).

[14] Karagiannis G., Altintas O., Ekici E., Heijenk G., Jarupan B., Lin K., Weil T. (2011). Vehicular networking: A survey and tutorial on requirements, architectures, challenges, standards and solutions. IEEE Commun. Surv. Tutor..

[15] Li Z., Wang J., Gao J., Li B., Zhou F. (2014). A vondrak low pass filter for IMU sensor initial alignment on a disturbed base. Sensors.

[16] Han S., Wang J. (2012). Integrated GPS/INS navigation system with dual-rate Kalman Filter. GPS Solut..

[17] Wang J., Lee H., Hewitson S., Lee H.-K. (2003). Influence of dynamics and trajectory on integrated GPS/INS navigation performance. J. Glob. Position. Syst..

[18] Li Z., Wang J., Li B., Gao J., Tan X. (2014). GPS/INS/Odometer integrated system using fuzzy neural network for land vehicle navigation applications. J. Navig..

[19] Zhang Y., Gao Y. (2008). Integration of INS and un-differenced GPS measurements for precise position and attitude determination. J. Navig..

[20] Farrell J. (2008). Aided Navigation: GPS with High Rate Sensors.

[21] Yang Y., Cui X., Gao W. (2004). Adaptive integrated navigation for multi-sensor adjustment outputs. J. Navig..

[22] Yang Y., Gao W. (2006). An optimal adaptive Kalman filter. J. Geod..

[23] Kealy A., Retscher G., Toth C., Hasnur-Rabiain A., Gikas V., Grejner-Brzezinska D., Danezis C., Moore T. (2015). Collaborative navigation as a solution for PNT applications in GNSS challenged environments–report on field trials of a joint FIG/IAG working group. J. Appl. Geod..

